# A Riemannian framework for incorporating white matter bundle prior in orientation distribution function based tractography algorithms

**DOI:** 10.1371/journal.pone.0304449

**Published:** 2025-03-25

**Authors:** Thomas Durantel, Gabriel Girard, Emmanuel Caruyer, Olivier Commowick, Julie Coloigner

**Affiliations:** 1 Univ Rennes, CNRS, Inria, Inserm, IRISA UMR 6074, EMPENN — ERL U 1228, Rennes, France; 2 Signal Processing Laboratory (LTS5), Ecole Polytechnique Fédérale de Lausanne (EPFL), Lausanne, Switzerland; 3 Department of Computer Science, Université de Sherbrooke, Québec, Canada; University of Minnesota, UNITED STATES OF AMERICA

## Abstract

Diffusion magnetic resonance imaging (dMRI) tractography is a powerful approach to study brain structural connectivity. However, its reliability in a clinical context is still highly debated. Recent studies have shown that most classical algorithms achieve to recover the majority of existing true bundles. However, the generated tractograms contain many invalid bundles. This is due to the crossing fibers and bottleneck problems which increase the number of false positive fibers. In this work, we proposed to overpass this limitation with a novel method to guide the algorithms in those challenging regions with prior knowledge of the anatomy. We developed a method to create a combination of anatomical prior applicable to any orientation distribution function (ODF)-based tractography algorithms. The proposed method captures the tract orientation distribution (TOD) from an atlas of segmented fiber bundles and incorporates it during the tracking process, using a Riemannian framework. We tested the prior incorporation method on two ODF-based state-of-the-art algorithms, iFOD2 and Trekker PTT, on the diffusion-simulated connectivity (DiSCo) dataset and on the Human Connectome Project (HCP) data. We also compared our method with two bundles priors generated by the bundle specific tractography (BST) method. We showed that our method improves the overall spatial coverage and connectivity of a tractogram on the two datasets, especially in crossing fiber regions. Moreover, the fiber reconstruction may be improved on clinical data, informed by prior extracted on high quality data, and therefore could help in the study of brain anatomy and function.

## 1 Introduction

Diffusion magnetic resonance imaging (dMRI) is an MRI modality that allows to measure the thermal agitation of water molecules in the brain [1]. This agitation being constrained by the tissues micro-structures, typically the nervous system axons in white matter, dMRI enables the voxelwise estimation of the orientations of the white matter fibers [2,3]. By randomly choosing seeds from within the brain white matter, then following, from one voxel to the next, the local fiber orientations, one can achieve to estimate the brain connectivity and characterize the physical connections that mediate information transfer between cortical regions [3]. This process is called fibers tracking, or fiber tractography, and the resulting set of white matter trajectories is called a tractogram [4]. The ability of those approaches to delineate the white matter fiber pathways offers an unprecedented insight into the structural connections of the human brain and an enormous potential for the study of human brain anatomy, development and function [5]. Moreover, tractography has been proven particularly useful to neurosurgeons for the planning of surgery, especially to preserve important white matter pathways during resections [6].

A multitude of new tracking algorithms have been proposed to improve the quality of the tractograms [7,8]. The first methods were deterministic methods, where only the principal diffusion orientation of the diffusion tensor was used in each voxel [9]. However, studies have shown the difficulties that these methods have to represent complex brain regions, like crossing or fanning fibers [10]. In response, new diffusion model, like the orientation distribution function (ODF) which encodes continuously all the diffusion orientations within a voxel and thus can characterize the complex fiber structure, have been developed [11,12]. Modern methods have then sought to estimate the fiber dispersion estimation using probabilistic methods [5,13,14].

Among them, existing local tractography approaches estimate the local fiber orientation at each voxel independently by simultaneously fitting the local model and propagating in the most consistent direction, such as Kalman filtering method [15], particle filtering method [16–18], random walks methods [11] and graph theory method [19]. Other approaches have proposed more global approach which computes multiple fibers pathways and select the best ones based on the diffusion characteristics, like using Bayesian model [20] or the Hough transform [21] and machine learning more recently [22]. However, despite advancements in dMRI data acquisition, as well as improvements in modeling and tracking of white matter fibers, certain limitations persist [23,24], hindering their extensive utilization in a clinical setting. A study conducted by Maier-Hein et al. in 2017 revealed that contemporary algorithms can successfully reconstruct up to 90% of the true white matter bundles. However, they tend to perform poorly in terms of accurately capturing the spatial extent of these bundles [24]. Moreover, those tractograms also contained large amounts of invalid bundles [24]. Overall, the iFOD2 [25] and PTT [26] algorithms had good performances in recent international challenges [24,27].

To overpass the poor spatial extent of white matter bundles reconstruction, one proposed solution is to increase the number of streamlines generated, leading to an over representation of easy to tract bundles (e.g. with a straight trajectory) and an under-representation of difficult to tract bundles (e.g. passing through complex white matter configurations, or with a high curvature), inducing a density bias [18,28]. As an illustration, local tractography techniques strive to deduce global connectivity based on local directional information. This approach can lead the algorithm to prefer the most straightforward path, even in intricate regions, which may not accurately reflect reality [24]. Various studies have suggested that advanced diffusion microstructure modeling [29,30], streamline filtering techniques [31–33] or advances in machine-learning-driven tractography [34] could reduce the false positive rate.

Other approaches have proposed to compensate the lack of global information by adding them in the form of anatomical prior, whose purpose is to guide the algorithm in complex regions. As for segmentation and label fusion approach, recent methods are based on the use of an anatomical atlas such as the TRACULA method [35], with promising results combining deterministic tractography and anatomical prior. Then, diffusion prior during the tracking process were proposed in [36–38], improving the delineation of white matter bundles. In [38], a bundle-specific method incorporates anatomical and orientational prior based on a template, to improve the reconstruction of long fibers, resulting in an increase of reproducibility, sensitivity and specificity. On the other hand, in [39], another approach based on a machine learning method was proposed to automatically segment, with high precision, the overall shape of a bundle.

Based on these promising results, we developed a method of anatomical prior creation and combination, which is usable with any tractography algorithm based on ODF [40]. Our method uses pre-segmented fiber bundles, as those generated by [39], to agglomerate global information from several different brains and capture the orientational variability in complex brain regions. In this work, prior are computed on this anatomical atlas and expressed in the form of voxel-wise TOD [41] and then combined with the ODF [40] data using a Riemannian framework [42]. We decided to incorporate those prior in two state-of-the-art algorithms using ODF data: MRtrix iFOD2 [25] and Trekker PTT [26]. We subsequently assessed these approaches by applying them to both the diffusion-simulated connectivity (DiSCo) dataset [43,44] and the Human Connectome Project (HCP) data (https://www.humanconnectome.org/). We also compared our method with two bundles priors generated by the bundle specific tractography (BST) method [38]. Our aim was to demonstrate the enhanced quality of the tractogram estimation, particularly with regard to the spatial coverage of the reconstructed bundles. An open-source implementation of the proposed method is available at https://github.com/Inria-Empenn/Anima-Public.

## 2 Materials and methods

**Fig 1:**
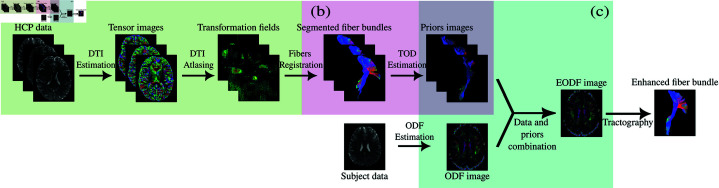
Overall method pipeline. (a) Construction of an anatomical atlas from segmented fibers. (b) Extraction and estimation of the TOD anatomical prior from the atlas. (c) Combination between the prior and the subject data using a Riemannian framework.

Our method can be separated into 3 distinct parts: i) the construction of an anatomical atlas from segmented fibers, considered as gold standard, ii) the extraction and estimation of the TOD anatomical prior from the atlas and, iii) the combination between the prior and the subject data. The entire framework is illustrated in Fig 1.

### 2.1 Atlas construction

The first step of our framework is the creation of a reference fiber atlas from a set of high-resolution diffusion images (see Fig 1a). In this paper, we chose high-resolution diffusion images from the HCP young adult study [45], which were acquired at high spatial resolution (1.25 mm isotropic) and three b-values with 90 diffusion-encoding directions each.

The proposed atlasing method, detailed in [46], follows a modified version of Guimond et al. (2000) [47] which was adapted for diffusion data, to compute an atlas of diffusion tensor images from a set of control subjects. This approach is based on a procedure which iteratively computes the atlas by registering the tensor images of HCP images onto a current reference. After each iteration, it performs an average of all the previous unbiased atlases to compute the next one at the following iteration. The main modifications from the original Guimond et al. (2000) method is to use diffeomorphisms encoded as Stable Vector Fields (SVF) and the log-Euclidean framework [48] to compute the average transformations and the approach was adapted to diffusion data.

For each of the 105 subjects from the annotated HCP data [49], 72 bundles were obtained using the TractSeg algorithm [39], which performs fiber tracking and filtering. This process involved initial filtering based on regions of interest (ROIs), followed by expert-guided refinement. The overall process used to segment these bundles is detailed in [39] and the data is available online [49]. The transformation field obtained by the atlasing method from each individual space to the atlas space was then applied to the 72 segmented fiber bundles of interest of each subject in order to all align them to the fiber atlas.

### 2.2 Extraction of the prior

The bundles of interest that were registered to a common space are combined in order to represent the general shape of bundles. In order to capture complex orientation, a local fiber orientation prior was estimated using the track orientation distribution (TOD) [41], in each voxel. The tractogram segments intersecting the voxel are represented as a probability distribution function (PDF) in the image domain, rather than a set of individual tracks (samples from this distribution). The TOD, thus, captures the expected fiber directions [41].

Our TOD imaging method operates as follows : first, all the fibers directions are extracted by calculating the local orientation of each fiber in the voxel. Then, the directions are clustered using a euclidean distance based *k*-means algorithm to define the main directions within the voxel and to correct for the density bias. This step is important to avoid over-represented fiber directions from contributing multiple times to TOD. The main directions are defined by the mean of the *k*-means clusters whose number range from 0, when no fibers have been detected, to 4, the maximum number of fiber crossings possible in a voxel, allowing us to characterize both complex crossing regions and simple linear ones. Then, the TOD in each voxel is represented using a set of modified spherical harmonics (SH) basis functions (see [40]) and constructed by projecting one spherical point spread function (PSF) per extracted main direction, then averaging these PSFs set.

The PSF, along the *z* direction, can be easily obtained in SH basis:


$$\begin{array}{c} \delta_{z}(\theta ,\phi )=\overset{\infty }{\underset{l=0}{\sum }}\overset{l}{\underset{m=-l}{\sum }}c_{l}^{m}Y_{l}^{m}(\theta ,\phi ), \end{array}$$ (1)

where 0 ≤ *θ* ≤ *π* and  − *π* ≤ *ϕ* ≤ *π* are the spherical coordinates, $Y_{l}^{m}$ the spherical harmonic of degree *l* and order *m* and $c_{l}^{m}$, the coefficients, given as:


$$\begin{array}{c} c_{l}^{m}=\int_{-\pi }^{\pi }\int_{0}^{\pi }\delta_{z}(\theta ,\phi )Y_{l}^{m}(\theta ,\phi )\sin(\theta )d\theta d\phi \end{array}$$ (2)

note that $c_{l}^{m}=0\text{ when }m\neq 0$ as the PSF is cylindrically symmetrical.

We found that resolving the with the PSF pointing towards the direction *θ* = 0 and then rotating them to match the direction extracted from the atlas greatly simplifies the calculations. In [50], authors showed a simple method to rotate functions expressed in SH basis. Since 4 different directions can be represented in a voxel, the final step of the extraction of the prior is to average the projected PSF. The averaging of distributions defined on the sphere is performed through a Riemannian framework [42]. To do that, we define $\psi (\theta ,\phi )=\sqrt{\delta_{z}(\theta ,\phi )}$, the square-root density function of the PDF *δ* ( *θ* , *ϕ* ) . The square-root is used to ensure that the logarithm maps are available in closed form. Thus, *δ* ( *θ* , *ϕ* )  has to be strictly positive. For this reason, in order to find the right PSF, several different distributions were tested (i.e. a Dirac distribution, a Watson distribution and a Gaussian distribution) and then were expressed on the SH basis. We found out that a 2D Gaussian distribution, defined on the unit sphere, is the only one whose number of negative values do not diverge with the degree *l*.

Then, in order to calculate the average distribution, we used the weighted Karcher mean, $\bar{\psi }$, of a set of $n\text{points}{\left\{\psi_{i}\right\}}_{i=0}^{n}$ in a Riemannian manifold defined by:


ψ¯= arg ⁡ min ⁡ ψ12∑i=0nωidist(ψ,ψi)2 (3)

with *i* ∈ [ 0 , *n* ] , $\omega_{i}\geq 0\text{ and }\sum_{i=0}^{n}\omega_{i}=1$. *dist* (*ψ*, *ψ*_*i*_) is the norm of the logarithmic map from *ψ* to *ψ*_*i*_, defined by:


log ⁡ ψ(ψi)=ψi−⟨ψi,ψ⟩ψ1−⟨ψi,ψ⟩cos ⁡ −1⟨ψi,ψ⟩ (4)

with  ⟨ . , . ⟩ , the normal dot product.

As described in [42], the unique solution is $\bar{\psi }$, such as:


∑i=0nωi log ⁡ ψ¯(ψi)=0 (5)

To estimate the prior, we calculate $\bar{\psi }$, which is the square-root of the prior for each voxel using the and uniform weighting $\omega_{i}=1∕n,\forall i\in \{1,...,n\}$, from $\psi_{i}$, representing *n* ( 0 ≤ *n* ≤ 4 ) different directions estimated initially by the *k*-means algorithm.

**Fig 2:**
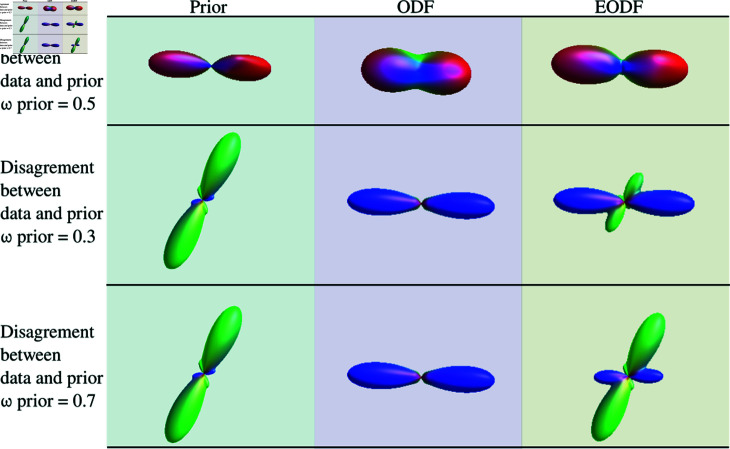
Examples of combination of the data and the prior. On the first row: prior and data are aligned. The EODF main orientation are unchanged. On the second and third rows: Prior and data are crossing. The corresponding EODF peak is more or less attenuated using the weight of the prior.

An example of the prior with the SH coefficients, truncated at a degree of *l* = 8, of the PSF described previously are shown in Fig 2. The prior creation process in order to average all extracted directions has to be calculated only once.

### 2.3 Combination of the data and the prior

As displayed in Fig 1, the ODF image of the individual diffusion dataset is calculated to estimate the next direction. During tractography, in order to inform the ODF with the anatomical prior,as explained in the previous section, a weighted Karcher mean is computed between the ODF and the prior using , to obtain the enhanced-ODF (EODF). However, this method involves calculating, for each voxel of the image, the dot product between distributions on the sphere, that implies an integral over the sphere which would complicate the process.

To simplify the computations, Goh et al. proposed to discretize the PDF and to work with the square root of histograms [42]. In this case, the dot product become summations.

Since the overall purpose of our method is only to guide the tractography algorithms and not to reflect the structure of the prior, the weighting factor $\omega_{i}$ must be well studied. We want to be able to distinguish between the simple linear regions, where not much guidance is needed, especially in easier-to-track bundles with only one fiber direction, and the complex regions with crossing fibers, where the use of the prior is more needed. In order to address this specification, we choose to use 2 measures to weight the prior: the generalised fractional anisotropy (GFA) [51], for the prior and the Akaike information criterion (AIC) [52] for the data.

The GFA, is given by:


$$\begin{array}{c} GFA=\sqrt{\frac{1-c_{0}^{2}}{\overset{N}{\underset{i=0}{\sum }}c_{i}^{2}}} \end{array}$$ (6)

where the $c_{i}$ are the SH coefficients. The GFA is a measure of the anisotropy within the considered voxel, that is, in the case of the prior, this value can reflect the complexity of a region.

The AIC, is described by;


$$\begin{array}{c} AIC=2k-2\ln(L) \end{array}$$ (7)

where *k* is the numbers of parameters in the model and *L* the maximized likelihood. The AIC is a measurement of the estimation quality of a model.

**Fig 3:**
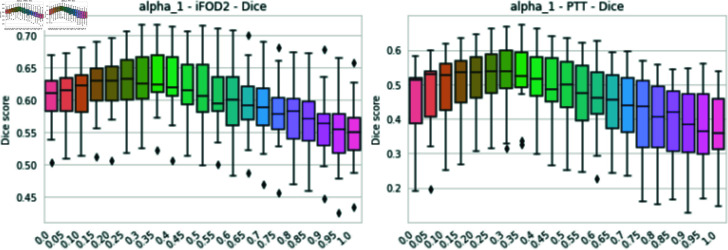
Dice score for various *α* values (from the Eq 8) for the tracking of the CST and for the iFOD and PTT algorithms. The method performs most effectively with an *α* value of 0.35.

Empirically, to well differentiate linear and complex regions, we defined the prior weight given by:


$$\begin{array}{c} \omega_{prior}=\alpha (1-GFA)+(1-\alpha )exp(\frac{AIC_{min}-AIC}{2}) \end{array}$$ (8)

where $AIC_{min}$ is the minimum AIC value measured over the entire image, *α* represents the amount of information extracted from the prior, via the GFA, and *β* 1 − *α* the amount of information extracted from the data, via the AIC. Those parameters need to be adapted to the data. For the experiments presented in this work, *α* is estimated using a study measuring the Dice score between the estimated fiber bundle and the ground truth and we choose *α* = 0.35 (see Fig 3).

**Fig 4:**
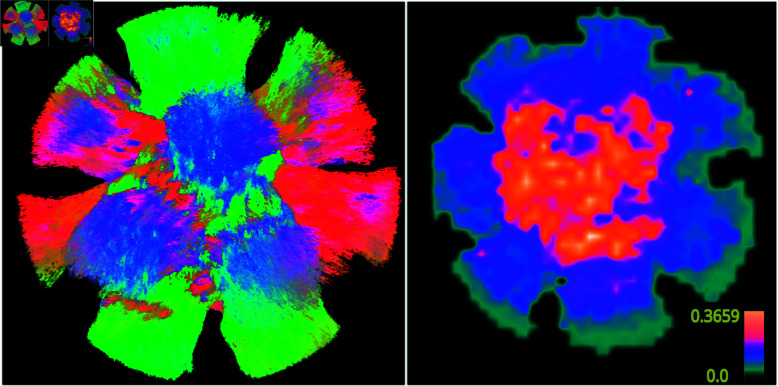
Left: DiSCo dataset pathways. Right: Prior weighting map example for the DiSCo dataset. Higher value denotes higher usage of the prior and thus more complex regions.

An example of a weighting map for the DiSCo dataset can be seen on Fig 4 with higher $\omega_{prior}$ in crossing fiber areas.

The result of this averaging process is then an enhanced-ODF (EODF), expressed in SH basis, usable in any ODF-based tractography algorithm. Fig 2 shows examples of combination of the data and the prior, as well as the effect of weighting on the results. The first row represents a voxel where the ODF and the prior have the same direction, corresponding to an easy-to-track regions with one-way crossing. On the other hand, in the second and third rows, the orientation of the ODF and the prior are different. In this case, according to the value of $\omega_{prior}$, the EODF is almost equal to the ODF or a mixture of the prior and ODF.

## 3 Experiments

In order to validate our method using prior, we conducted our first experiments on the DiSCo challenge dataset [27]. Then, we tested our algorithms on HCP data. For each experiment, we implemented our prior approach on two state-of-the-art algorithms, MRtrix iFOD2[25] and Trekker PTT [26]. Thus, on the two dataset, 4 tractography reconstruction were performed, both with and without using prior. For iFOD2, we used a step size of 0.2 voxel and a maximum angle between successive steps of 20 degrees. For PTT, we used the default parameters, that is, a step size of $\frac{1}{40}\text{ and a minimum radius of curvature of }\frac{1}{2}$ of the smallest voxel dimensions.

### 3.1 DiSCo challenge dataset

The aim of this experiment is to test the ability of our novel method to improve the connectivity estimation and the effect of noise.

In this context, we do not have access to several datasets to build an anatomical atlas, instead, we computed the TOD: (i) using the ground-truth fiber pathways as anatomical prior and (ii) using fiber tracked on high quality data as anatomical prior.

We performed two different variants of this experiment: in the first one, prior are estimated on ground-truth fibers, not available on in vivo data; and the second experiment is used to demonstrate the benefit of our method, without having a ground-truth but prior build on high resolution data.

*(i) Fiber pathways ground-truth:* The DiSCo dMRI images [43] have a grid of 40×40×40 with voxel size of 1 mm^3^ and are acquired with 4 different b-shell of 1000, 1925, 3094, and 13191 s/mm^2^, 90 directions per b – –shell and 4 b0. In order to obtain images with a quality comparable to clinical data, we used downsampled data with a grid of 20×20×20 voxels and only kept the 1925 s/mm^2^ b-shell with 90 directions. The prior is computed with the original data. In addition, to study the effect of noise, the dMRI was corrupted using various levels of Rician noise according to the method described in [43]. The resulting images have an average SNR of 0, 10, 20, 30, 40 and 50. The individual ODF were computed using the method described in [53] and represented in a spherical harmonic basis truncated at the 8^th^ order. Then, EODF were calculated using the method described in Sect 2.

*(ii) multi-shell ODF:* In this second set of experiment, we computed the prior on fibers tracked with the multi-shell multi-tissue constrained spherical deconvolution (CSD) fiber ODFs. The idea is to simulate a clinical context by computing the ODF on clinical dMRI data and combined with anatomical prior calculated on high resolution data. The anatomical prior are calculated on 40×40×40 voxels DiSCo dMRI at SNR 20. From those images, fiber ODF were computed using the MRtrix implementation of the multi-shell multi-tissue constrained spherical (msmt-CSD) method described in [54] using default parameters and using 8^th^ order SH basis. Then, fibers were tracked using the MRtrix iFOD2 algorithm with a step size of 0.2 voxel and a maximum angle between successive steps of 20 degrees. Only the streamlines that reaches the endings ROIs were kept. Finally, the TODs were extracted from those fibers using the method detailed in the previous section. After the estimation of the prior on high resolution data, we estimated the ODF of a subject data with 20 × 20 × 20 *grid and SNR* = 20. Then, EODFs were obtained by combining the TOD with the clinical data fiber ODF.

To quantify the results, we computed the Pearson correlation coefficient (r) between the ground truth connectivity matrices of the three DiSCo datasets and the resulting tractograms connectivity matrix [44]. These matrices are computed by counting the numbers of streamlines that reach both start and end ROIs for each DiSCo fibers bundles.

### 3.2 HCP data

To test our method on in vivo high quality data, we used the HCP young adult data, acquired with 90 gradients distributed on 3 shells of b=1000, 2000, and 3000 s/mm^2^ with 6b = 0 acquisitions and a final resolution of 1.25*mm*^3^.

Among them, the 105 pre-segmented HCP images were used, as described in Sect 2.1, to build fiber atlases of the Arcuate fascicle (AF), the Cingulum left (CG), the Corticospinal tract (CST), the Optic radiation (OR) and the Superior longitudinal fascicle I (SLF I) to study the influence of the addition of prior on bundles of different degrees of complexity and in different regions of the brain. The atlas bundles segmentation are defined in [39] and are available online (https://zenodo.org/records/1477956). We employed the RecoBundles algorithm [55] with our atlases as models to extract streamlines from the whole brain tractograms for the data bundles. A cross validation was performed on the 100 subjects are used to build the atlas and the 5 remaining to perform the tractography algorithm. This process is repeated 8 times, giving us a total of 40 subjects to test the method on.

For each image belonging to the training set, ODFs were computed on the shell b=3000 s/mm^2^ using the method described in [53] and represented in a spherical harmonic basis truncated at the 8^th^ order. Then, the TOD images for each bundle were registered on the subject space. Those prior were incorporated to the data before the tracking process for each subject. The tractography algorithms were tested on the 5 remaining subjects.

In order to compare our method with state-of-the-art anatomical prior methods, we used the BST algorithm [38], using the default parameters, to compute bundle specific priors for the CST and the AF bundles. We then tracked those bundles with the iFOD2 and PTT algorithms using the same parameters as before. To segment these bundles from the whole brain tractogram, we utilized the RecoBundles algorithm [55] alongside our atlases as models. Subsequently, for ensuring accurate connectivity of streamlines to their respective regions, we relied on the end-points segmentation derived from the TractSeg algorithm [39].

To quantify the overall shape quality of the tractograms, we computed the generalized Dice score [56] between the segmentation of fibers obtained with tractography algorithm and the reference fibers. In order to measure the improvement on the numbers of streamlines reaching both ROI endpoints, we computed the percentage of streamlines that correctly connects the beginning and the end regions of bundles over the total number of streamlines in the reference bundle (noted valid streamlines VS). It is worth noting that this measure does not mean anything in absolute, being biased by the numbers of streamlines in the reference tracks. However, since the same numbers of streamlines is generated for each bundle, it allows us to compare the overall bundles shape and quality with and without the addition of prior.

## 4 Results

### 4.1 DiSCo challenge dataset

**Fig 5:**
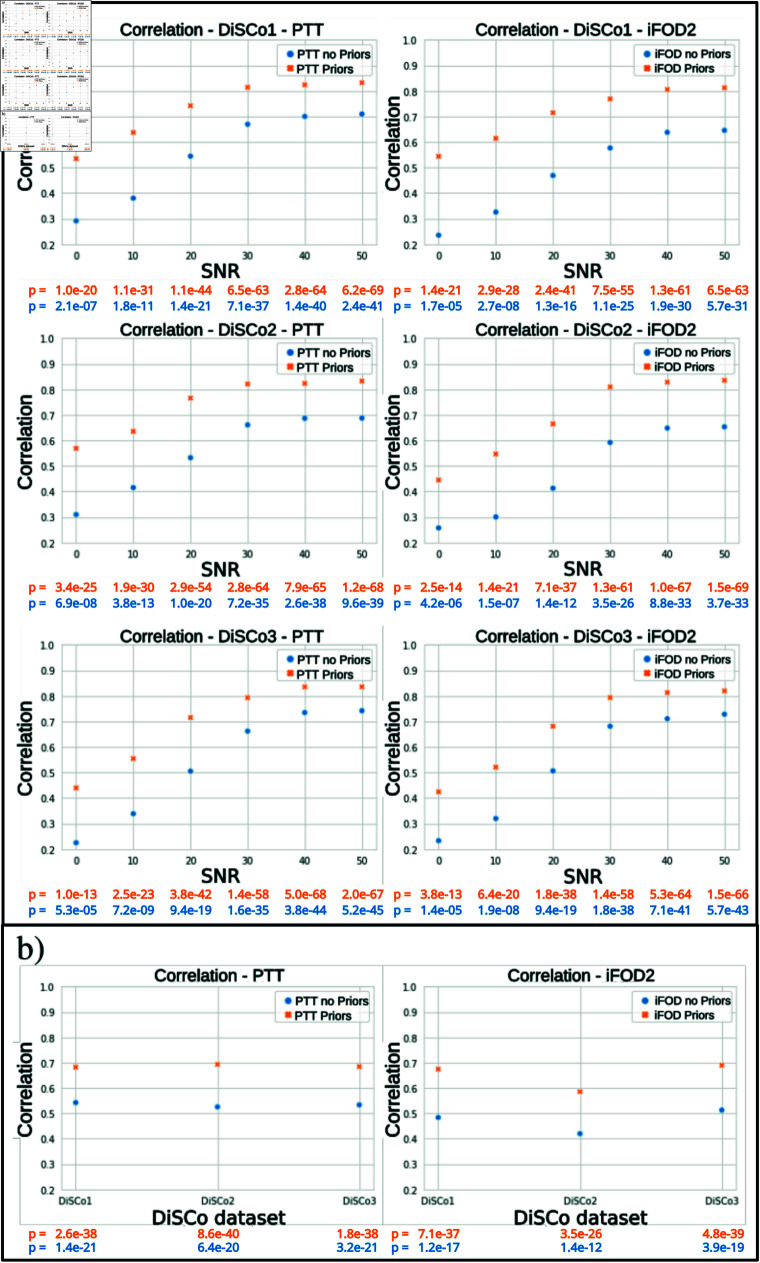
Pearson correlation for the DiSCo experiments: (a) Fiber pathways ground-truth experiment with respect to SNR (b) Multi-Shell ODF experiment for the three DiSCo datasets. First column for PTT and second for iFOD2. In all cases, blue points denotes measure without prior and the oranges ones with prior.

Fig 5 displays the correlation with the ground-truth connectivity of the two experiments, as well as the adjusted statistically significant p-values for multiple comparisons using the Bonferroni method (*n* = 6), for the three DiSCo datasets. First, on Fig 5a), we observed that the lower the noise is, the better are the correlation improvement for the experiment using the fiber pathways as ground-truth. Next, both algorithms performed better with the addition of prior for all level of noise and the three datasets. Additionally, it’s worth noting that the enhancement diminishes as data quality improves. On average, the correlation increases by 0.23with an SNR of 10 dB, but only by 0.11 with an SNR of 50 dB. Furthermore, the mean correlation improvement is 0.19 for the iFOD 2 algorithm and 0.17 for PTT. Finally, the average maximum correlation achieved is *r* = 0.83 for PTT and *r* = 0.82 for iFOD2. There are no major differences between the three datasets. For the second experiment using high resolution DW-MRI data, we showed an average correlation improvement of 0.15 for the PTT algorithm and 0.17 for the iFOD2 algorithm, in Fig 5b). Incorporating anatomical prior calculated on high resolution data during the tracking process of low resolution data increases the Pearson correlation coefficient with the ground-truth connectivity matrix.

### 4.2 HCP data

**Fig 6:**
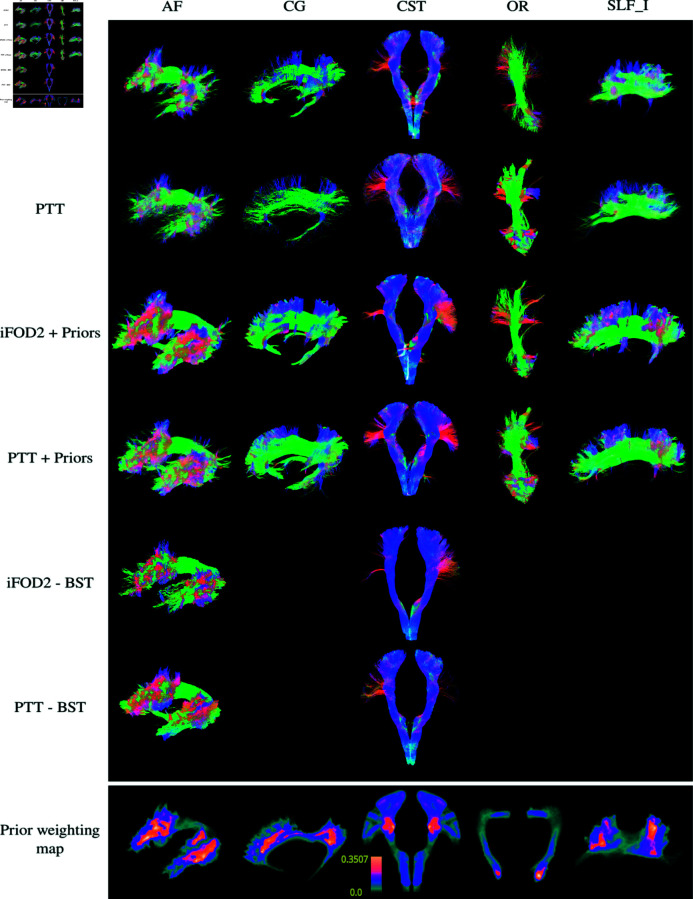
Visual comparison of the resulting tractograms. From top to bottom line: iFOD2, PTT, iFOD2 with prior, PTT with prior, iFOD2 with BST priors and PTT with BST priors. Last line: Prior weighting map, where brighter pixel denotes a higher usage of the prior. From left to right: The Arcuate fascicle (AF), the Cingulum (CG), the Corticospinal tract (CST), the Optic radiation (OR) and the Superior longitudinal fascicle I (SLF I). Note that the quantitative results presented in the rest of the work use both sides of each bundles. But to simplify visualization, only one side is shown here for all bundles, except for the CST.

**Fig 7:**
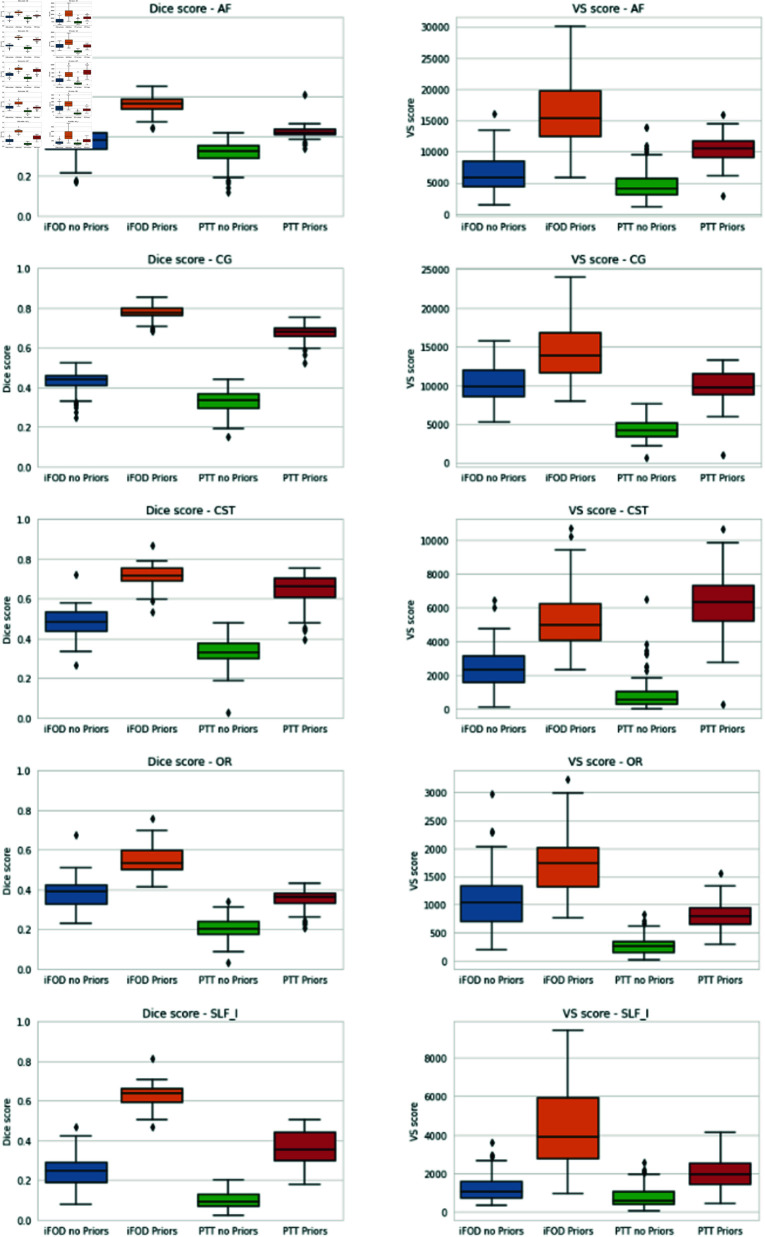
On the left, Dice score result for the HCP experiment. On the right, Valid Streamline (VS) score result for the HCP experiment. From top to bottom : The Arcuate fascicle (AF), the Cingulum (CG), the Corticospinal tract (CST), the Optic radiation (OR) and the Superior longitudinal fascicle I (SLF I).

**Fig 8:**
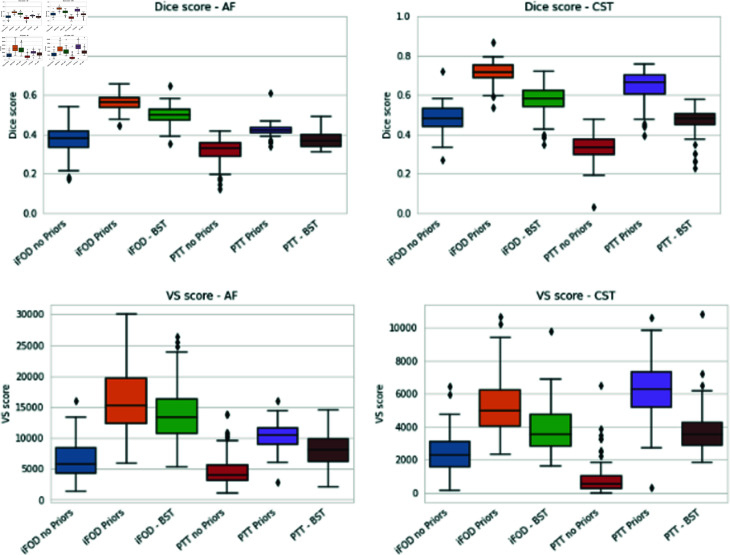
Comparison with the BST algorithm for the AF and CST bundles. The top row present the Dice score and the bottom row the VS numbers. In average, our method preforms better for both measures.

Fig 6 displays the segmentation of AF, CG, CST, OR and SLF I, obtained with iFOD2 and PTT, with and without prior and the segmentation of the AF and CST obtained with the BST method. The results for the Valid streamlines and the Dice score are presented in Fig 7. For the Valid Streamlines (VS) score, the addition of prior appears to always increase, on average, the numbers of streamlines that connect both end regions. However, enhanced tractography appears to augment the variability of the outcomes, as shown by a mean variance across all bundles of 7.19% without prior and 19.84% with prior inclusion. We also noticed that incorporating the prior in the PTT algorithm improves more the Valid streamlines than with the iFOD2 algorithm. Indeed, the average improvement for iFOD2 is of 25.08% and 31.37% for PTT. The same observation can be made for the Dice scores, but as opposed to the Valid streamlines, the variability seems to decrease with the addition of the prior. We notice that incorporating the anatomical prior improve the spatial coverage and that a higher fraction of streamline reach the endpoints of the bundle. In Fig 8, a comparison is presented between our approach and the BST method concerning AF and CST bundles. The visual representation demonstrates that using our priors yields a more substantial enhancement in fiber estimation, as indicated by both the Dice score and the number of valid streamlines, compared to the BST method.

## 5 Discussions

In this work, we proposed a novel method for creating and incorporating anatomical prior to any ODF-based tractography algorithms. We showed that incorporating anatomical prior on two state-of-the-art tractography algorithms improves the overall quality of tractograms when prior are extracted from a ground truth, but also from high quality tractograms. Indeed, on the DiSCo dataset, the proposed prior-based tractography methods obtained better correlation scores between the reconstructed bundles and the ground truth for every level of noise than the standards probabilistic algorithms, but this improvement reduces with the increasing SNR. Thus, the addition of prior to ODF images improves the quality of tractograms particularly in the presence of noise. However, it should also be noted that this improvement seems to decrease when the SNR of the data is increased.

More particularly, in the DISCO experiment, we tried to mimic real clinical context with low spatial resolution data and an SNR of 20. In this case, our results showed a better correlation for two methods incorporating prior between the estimated fiber connectivity and the ground-truth, reducing false positive fibers. This could allow us to consider using this method on clinical data of average quality, informed by prior extracted on high quality data. On the HCP data, the same results were obtained with an increase of the fraction of valid streamlines numbers when anatomical prior were incorporated during tracking process. In the same time, due to the increase of the Dice score when adding prior, the overall shape of the bundle seems to be also modified. We also reported an increase in variability across subjects of the tractography with prior compared with the ones without prior.

The effect of the prior seems to be more effective in complex fibers configurations, like fanning and crossing fibers or in regions of high fiber curvature. Indeed, our method is able to better delineate and estimate precise details. For the CST, it is well known that the upper part, that fans into the cortex, is hard to estimate. Without prior, the iFOD2 and PTT algorithms only manage to estimate a portion of the CST. While, with the addition of anatomical prior, they achieve a more complete reconstruction of the fanning portion. Another example is the OR bundle, where is the Meyer’s loop, with its highly curving fibers, is still a tractography open challenge [57]. When guided by EODF, both tested algorithms show results that, while containing spurious streamlines, allow a better recovery of the anterior extent of the Meyer’s loop. This result can be easily explained by the fact that in regions with crossing fibers, more information is taken from the prior. See the right column in Fig 6 where the brightest regions correspond to the most complex fibers microstructure regions and denotes a higher usage of the prior.

In addition, the use of AIC in the averaging process serves as a measure of "confidence" in the subject’s data estimate. This approach ensures that the atlas is only incorporated when the subject’s data alone cannot provide satisfactory results. By employing this weighting factor, the amount of information drawn from the atlas remains constrained, thereby preserving the specificity of the subject. Consequently, when the subject’s model is accurately estimated, minimal or no reliance on the atlas occurs, thereby maintaining individual subject variability. This assertion is supported by two observations: firstly, in Fig 5, where it can be observed that the variability of results after the addition of prior remains largely unchanged on average, and secondly, in Fig 6, where the last line illustrates weighting maps indicating the weight of the atlas, which never exceeds 0.35.

It is important to note that the results presented in this paper use iFOD2 and PTT algorithms as proof of concept, but the the proposed method aim at improving the spatial coverage and the numbers of streamline reaching the endpoints of the bundle of any ODF-based tractography algorithm. In fact, future works should study the contribution of prior addition with other algorithm but also generalize this method to other diffusion model.

In [38], authors generated sharp TOD by projecting Dirac in the SH basis. However, our experiences have shown that this also generates a number of negative values which increases with the truncated SH basis order, due to the Gibbs phenomenon. Thus, to overpass this problem and remove the negative values, they use apodized delta function [58] proposed in [41], but at the cost of a loss of angular resolution. Using this PSF, we would have to truncate at a higher order the TOD SH basis to obtain the same angular resolution that we have with the $8^{th}$ order of the Gaussian PSF [41]. So we decided to keep the Gaussian PDF and the ODF estimated without CSD, at the expense of less sharp ODF peaks, our interest here being to compare the tracking connectivity with and without prior. In the same way, in [38], prior and data are combined by an element-wise multiplication between the two sets of SH coefficients, followed by a normalization. However, our approach (i.e. using a Riemannian framework), allows us a better control over the weighting between the TOD and ODF and also enables, for future works, more complex operation on TOD, such as interpolation between different set of prior or even TOD filtering to remove unnecessary information, in order to improve the prior quality, at the cost of more approximation during the TOD estimation and an increased complexity.

Other works that studied the idea of guiding tractography algorithms, like [35], [36], [37] or [38], use non-linear registration and atlasing techniques during the atlas, or template, creation process, preferring to do an average of the references images. This could smooth the variability across the atlas and could also be the source of errors that would be accumulated in the final atlas and would, in the end, inject a bias inherent to the atlas in the tractography process. In our approach, using a modified Guimond method [47], the reference images are, at each step, iteratively registered with a non-rigid transformation onto the current space that become the reference space in the next step ant it is only the average of the transformation that is computed at the end of each step. With this approach, all the variability information is accumulated in the atlas and the errors are not included in the final atlas. Thus, no bias is introduced in the tractography.

In this study, the prior is estimated using uniform weighting in on bundle crossings, which may also introduce bias to tractography results. However, we demonstrated a significant improvement in fiber reconstruction quality despite this bias. In future works, we may consider to adapt those weights using the COMMIT algorithm [31].

Also, in order to study the global connectivity of the brain, our method also permits, by concatenating the individual bundles prior or by using a whole brain tractogram as prior, the construction of full brain atlases, and thus enable guidance of whole brain tracking. Therefore, it could be used for clinical studies where prior would be constructed from high resolution dMRI data in order to guide tractography algorithm on poor, clinical, resolution data.

Finally, our method has proven to be able to increase the quality of the estimated fibers, in term of spatial extent and number of valid streamlines when utilized on healthy subjects. These promising results could already enable advances in an academic context for the study of the healthy brain. In a clinical context, if used on patients suffering from pathologies that only slightly or moderately modify the white matter, such as psychiatric pathologies [59], this method could also bring improvements in fiber estimation, perhaps through a more in-depth study of the *α* parameter. Research along these lines should be pursued in future work. However, when working on patient suffering from severe brain alteration, such as stroke, this method could produce a tractogram solely guided by the prior in the affected regions, thus removing the specific microstructural modification and therefore may not be the most appropriate approach. In such scenarios, a solution might be to import other types of information into the prior. Either in the way the data and prior are weighted, using for example Apparent fiber density [60] of the data to potentially detect these brain modifications and thus prevent the fibers from being tracked solely on the prior, or directly in the estimation of the prior using other imaging modalities, such as myelin-sensitive relaxometry [61] in order to obtain prior images describing these brain regions in greater detail.

## 6 Conclusion

In this paper, we developed a novel method of anatomical prior creation and combination, which is usable with any ODF-based tractography algorithm. The prior are computed on fiber atlases and expressed in the form of TOD, in order to characterize the white matter microstructural variability across several brains and multiple fiber directions. Then, our prior is incorporated to guide the ODF-based tractography algorithm. Based on our results on DiSCo and HCP data, incorporating our anatomical prior improves the fiber reconstructions, in terms of spatial extent and valid streamlines, especially in crossing fiber regions. Furthermore, we have shown that, on the data used in this study, our method outperforms previous methods using anatomical prior in the tracking of the CST and the AF. Moreover, our approach could also enhance the tractography in the context of clinical data, by incorporating prior estimated on high quality data, which could help for the study of neurological diseases. The code, along with instructions for running it, is available at https://github.com/todurante/Durantel_PlosOne_2024.
